# Fracture resistance of different overlay designs with novel lithium disilicate materials on 3D printed dies

**DOI:** 10.1186/s12903-025-07629-0

**Published:** 2026-02-01

**Authors:** Radwa Salah Atta, Ahmad Aboelfadl, Nancy Essam Bahig Rafla

**Affiliations:** Department of Fixed Prosthodontics, Faculty of Dentistry, Ain Shams University, Organization of African Unity Street, Heliopolis, Cairo, Egypt

**Keywords:** CNC preparation, 3D printed dies, CAD/CAM, Lithium disilicate, Minimally invasive restoration, Fracture resistance

## Abstract

**Statement of problem:**

Moderate coronal destruction cases have recently been repaired using minimally invasive adhesive overlays with varying cavity depths and occlusal preparations. The optimal cavity preparation (criteria and dimensions) varies according to clinical crown length, patient age and inter occlusal space. Consequently, design and thickness of restoration will affect esthetics, function and fracture resistance necessitates the employment of new materials capable of meeting their full potential. However, there are numerous available materials and different preparation designs. However, there is insufficient data on the selection of appropriate material, preparation thickness and their effect on fracture resistance.

**Objective:**

To evaluate the fracture resistance of chairside (CAD/CAM) lithium disilicate using two diverse overlay restoration designs for premolars.

**Materials and methods:**

Specimens were prepared using a CNC milling machine, according to two different overlay designs: (A) overlay with 2 mm cavity depth & 1.5 mm occlusal thickness, (B) overlay with 1.5 mm cavity depth & 2 mm occlusal thickness. 42 restorations were designed and fabricated with a chairside CAD/CAM system (Mc XL, Dentsply Sirona) using 3 CAD/CAM lithium disilicate blocks (IPS e.max CAD, Amber Mill, CEREC Tessers) (14 specimens/group). Restorations were luted to 3D printed resin dies using standard resin luting cement (Breeze, Pentron) and then loaded with a steel indenter until fracture. The group findings were analyzed using one-way analysis of variance, and the medians were assessed independently using Kruskal-Wallis. The null hypothesis states that there will be no significant difference in the fracture resistance between the three CAD/CAM lithium disilicate materials and the two preparation designs.

**Results:**

The fracture force of CAD/CAM lithium disilicate restorations varied significantly based on the restoration design. Among the two overlays, the restorations with design A showed significantly higher fracture force than the restorations with design B (*p <* 0.001). Within design A: there was no significant difference between the materials. Within design B: there was a significant difference in fracture resistance between materials.

**Conclusions:**

Design A, including more cavity depth (2 mm) related to less occlusal reduction(1.5 mm) provides more fracture resistance values than design B using lithium disilicate overlay preparation design. Preparation design has a great effect on fracture resistance values of overlay restorations, while material type has negligible effect. Despite the presence of additional virgilite crystals in CT, it did not produce any increase in fracture resistance.

## Introduction

A conventional full-coverage crown preparation requires the removal of sound tooth structure ranging from 67.5 to 75.6% [1]. This aggressive preparation compromises the amount of remaining dentin support and pulp vitality; thus, it should be indicated when destruction is severe, especially when tooth structure loss is more than 50% [2]. In cases of less extensive tooth defects, more conservative preparations have shown to be advantageous [3]. In contrast, partial coverage preparations reduce tooth structure removal to a range of 35.5–46.7% only [1].

### Minimally invasive preparation designs

As the tooth structure becomes substantially destructed, recreating proper contours, contact and occlusion via a direct restoration becomes more challenging. Hence, indirect restorations are considered [9].

Subsequently, preparation for indirect restorations must be carefully designed to cover the weak structure with no undercut integration. The final restoration is required to fit passively [10], and is commonly retained by the efficiency of either luting cement or an adhesive system [11].

Overlay is an indirect restoration that covers all cusps when the isthmus of a mesio-occlusal-distal (MOD) restoration involves more than half the crown’s bucco-lingual width [12]. In literature it can be referred as an onlay with buccal or/ palatal extension.

The preparation design should retain the residual tooth structure and offer sufficient material thickness in order to reduce the risk of crack formation and ensure long-term durability of ceramic restorations [13].

### Ceramic materials

Recently ceramic materials have been evolving, with lithium disilicate being the most popular with different restorative designs.

LDS glass ceramics are primarily advantageous for esthetic restorations in the anterior area [14], and also due to their phenomenal biomechanical characteristics, they can be used as monolithic overlays in the posterior area [15].

lithium disilicate present commercially in block form under 3 different brands which are e-max CAD, CEREC Tessera and Amber Mill. They differ according to substructural size and amount of fillers which affect their physical properties.

E-max CAD is microcrystalline LDS with 70% lithium disilicate creating a 360 MPa flexural strength [16–18], while Amber Mill and CEREC Tessera are nanocrystalline LDS where crystals present in numerous tiny phases. In addition CEREC Tessera contains virgillite crystals that pushes its axial flexure strength beyond 700 MPa [19].

In contrast to IPS e.max CAD or Amber Mill, which need to be crystallized, CEREC Tessera merely needs an extra glaze fire (matrix firing) lasting 4.5 to 12 min at 760 °C, which can boost the material’s strength [20].

#### Fracture resistance

Ceramic restorations, like inlays or overlays, rely primarily on their fracture resistance to guarantee their longevity and durability. Factors influencing clinical fracture failure in ceramic restorations including cavity design (or preparation design), mechanical characteristics, restoration thickness, cementing agent, amount of functional load, and internal flaws [21–23].

## Material & methods

### Specimen Preparation

**Fig. 1:**
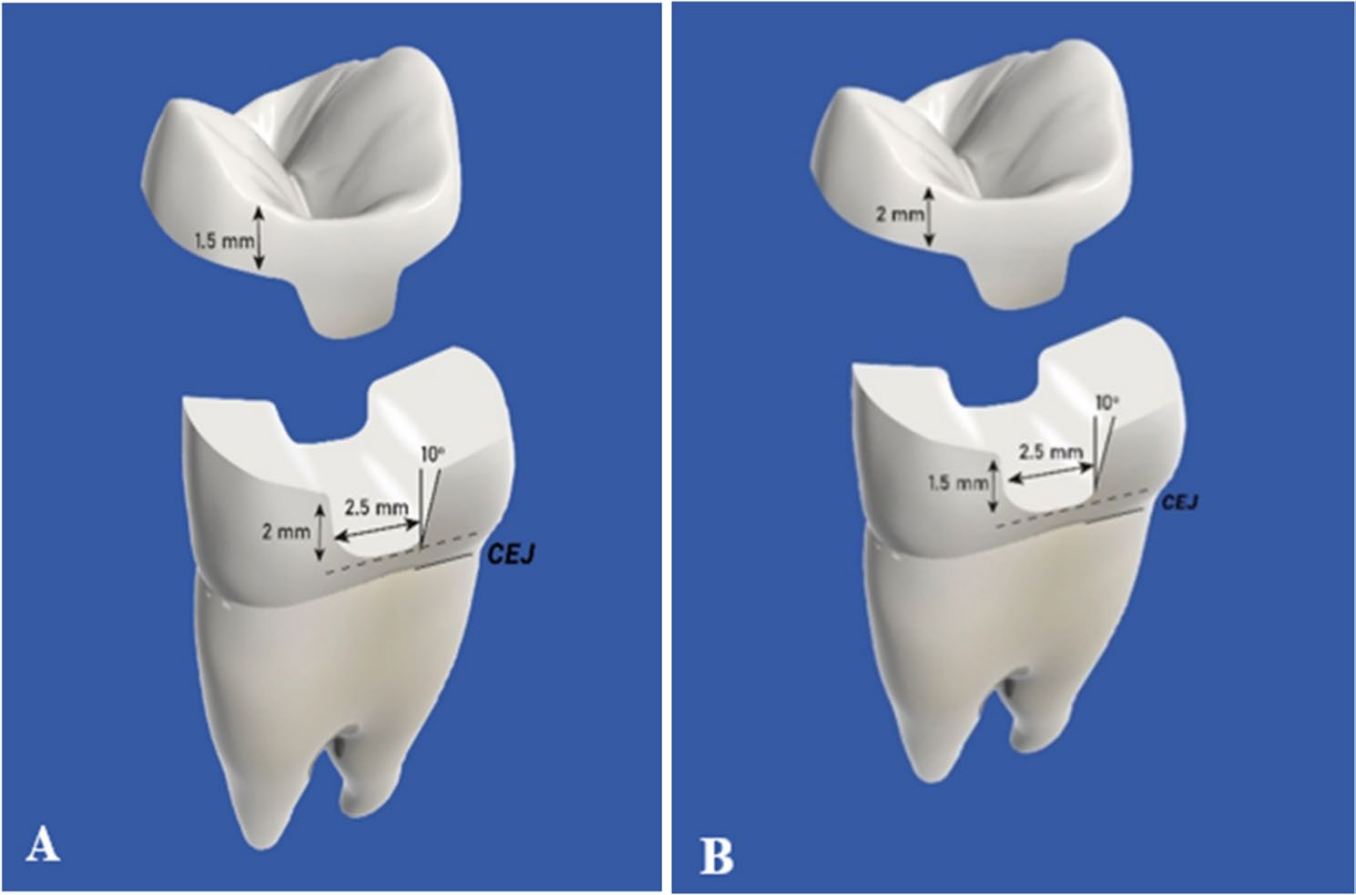
Diagram showing both designs

**Fig. 2:**
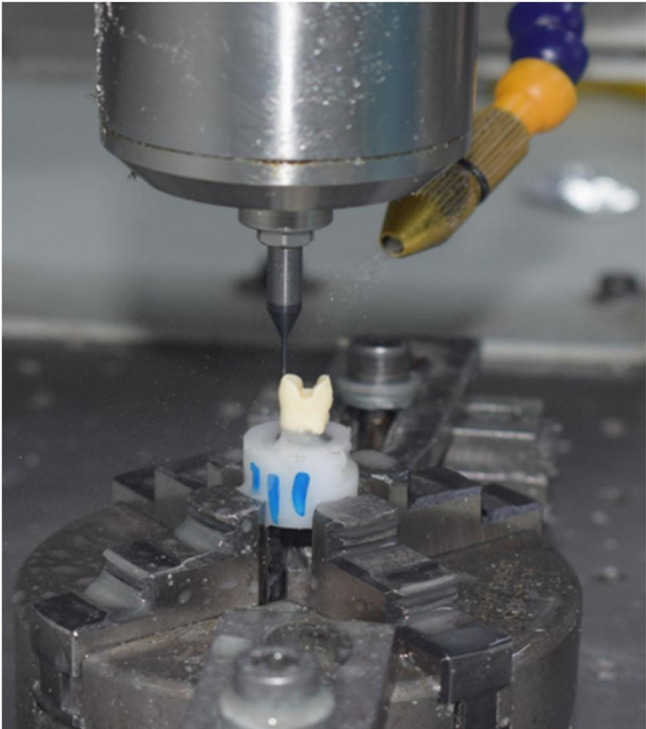
Tooth cutting by CNC machine

Two typodont acrylic maxillary premolars teeth were placed upright in molds filled with non-shrink epoxy resin material using a surveyor. They were fixed to the CNC machine to make a notch in a specific site facilitating the relocation of teeth later. Typodont teeth were scanned using an intraoral scanner for designing the preparation. Each Preparation was designed first on (Autodesk Inventor Professional) software with an anatomical occlusal reduction generating either 1.5 or 2-mm clearance for the overlay and butt joint finish line, followed by pulpal retention cavity extending either 1.5–2 mm from the central groove and 2.5 wide mesio_occluso_distal slot (occlusal isthmus) with non-proximal boxes. Cavity should have 90° Cavo surface angle, 10-degree divergence of the walls towards occlusal surface, rounded internal line angles, no bevels, and no undercuts (Fig. 1) A: for design (A),B: for design (B). Cutting teeth was performed using a Computerized Numerical Control (CNC) milling machine (ECNC 5030 M, EMAR) for the purpose of standardization (Fig. 2).

### Specimen duplication

**Fig. 3:**
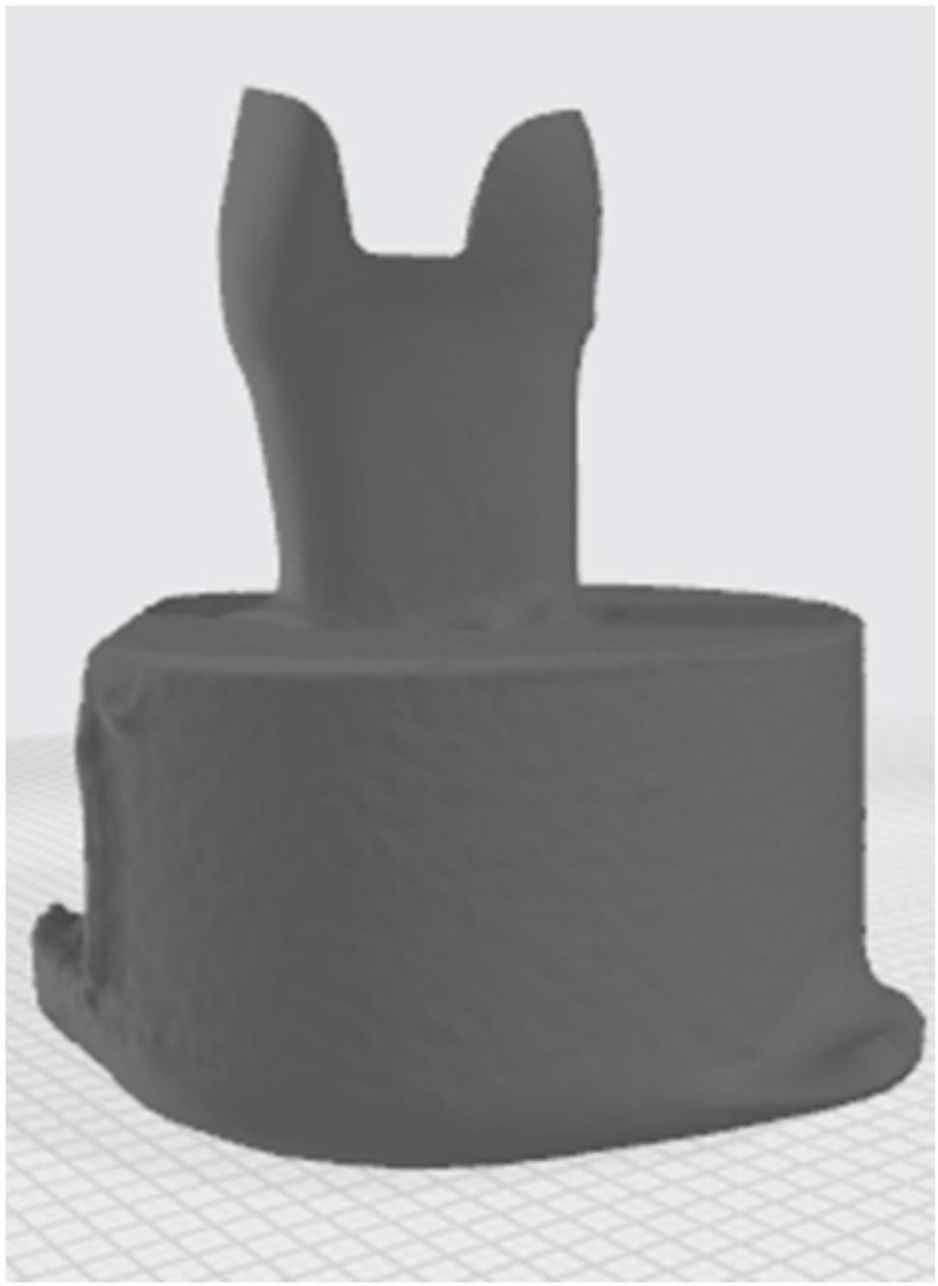
Scanning of prepared specimen

**Fig. 4:**
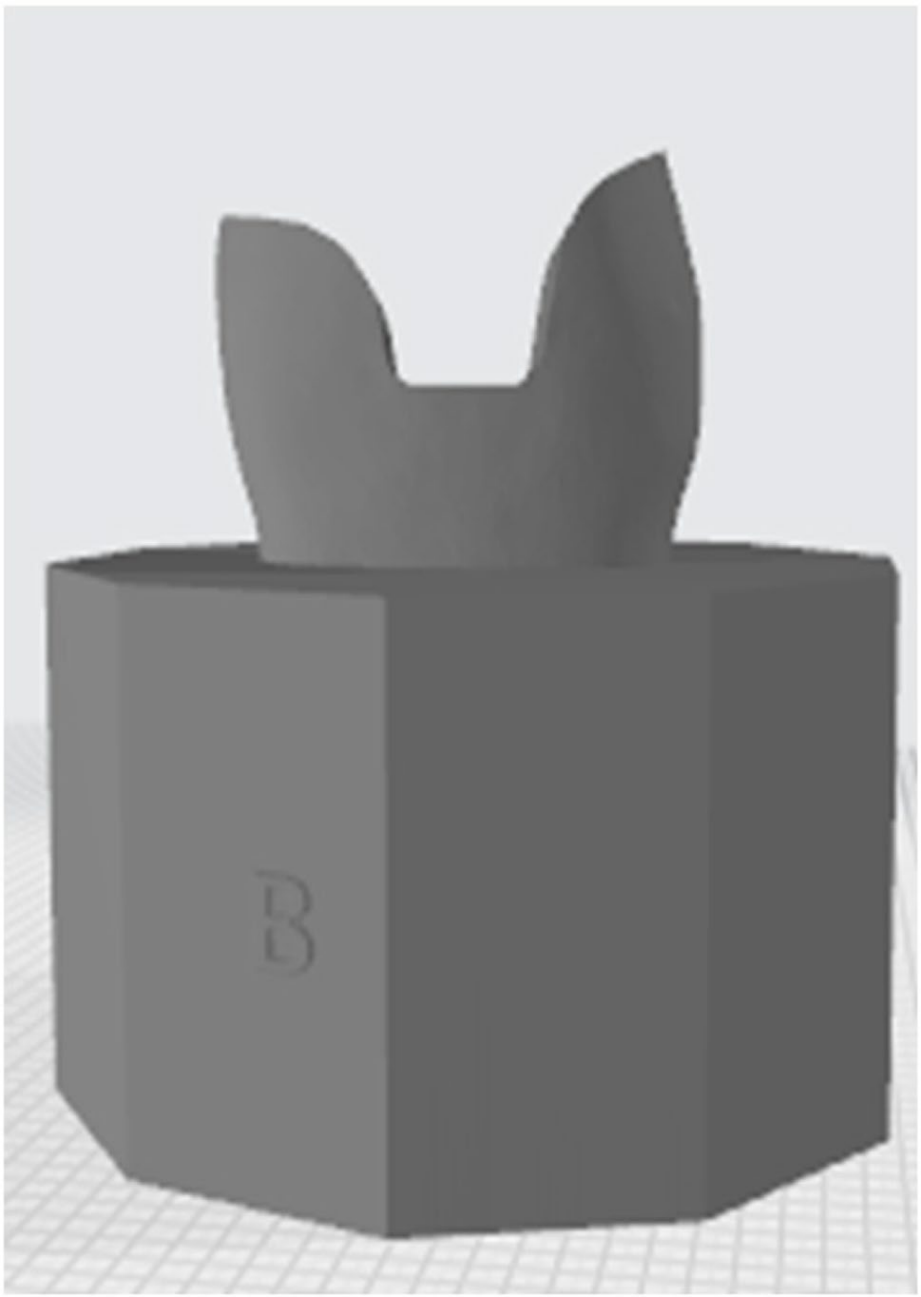
Modifications for 3D printing

The prepared acrylic teeth were scanned using a LED intraoral scanner (DIOS 4.0, i500, MEDIT, KOREA) starting with the occlusal side of prepared tooth then moving to proximal side followed by rotation all around (Fig. 3).The two STL files of prepared tooth were modified using Model Builder software (3D builder, 20.0.3.0, Microsoft Corporation) to simulate the bony support, exposing 2 mm of root surface below the cementoenamel junction and a maximum of 15 mm in diameter of the base to fit in the testing machine. Forty-two dies were printed from 3D printing resin (twenty-one die from each prepared tooth), which has the following physical properties after curing (Bending modulus: 1.192–2.525 Mpa, Density: 1.05–1.25 g / cm3, Modulus of elasticity: 10 GPa) which is close to modulus of elasticity of dentin (15 GPa approximately), using a laboratory printer (ELEGOO Saturn, CHINA) with UV resin photocuring technology (Fig. 4).

**Fig. 5:**
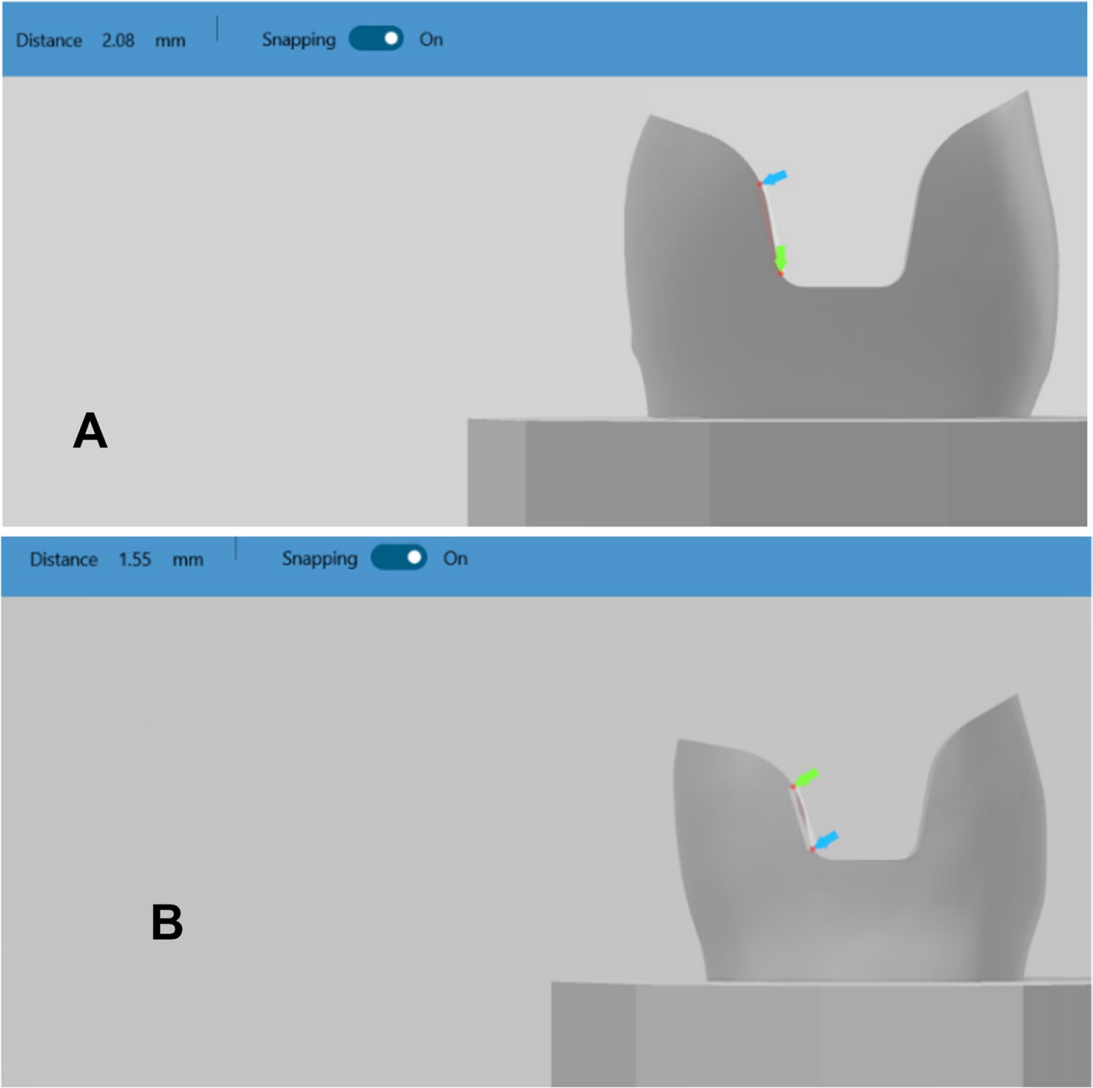
Virtual measurement of both dies

Checking and verification of standardized preparation among each subgroup through virtual measurements (Fig. 5) A: for design (A), B: for design (B).

### Samples fabrication

**Fig. 6:**
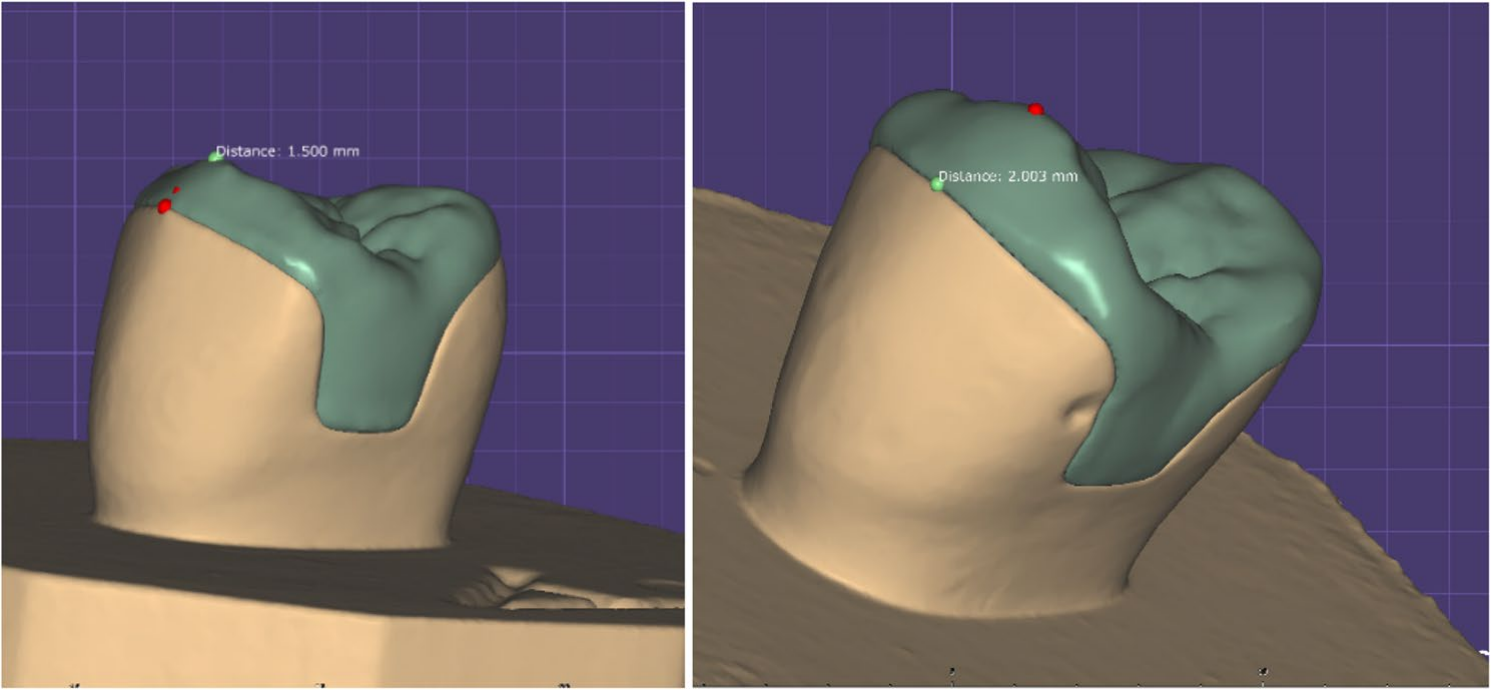
Samples thicknesses at cusp tip

Designing the restoration was done using Exocad Galway 3.0 software (Fig. 6). All specimens were fitted with the anatomy of the second maxillary premolar. Forty-two ceramic restorations were milled using Mc XL milling machine. The combination (crystallization/ glaze) was conducted in a compatible ceramic furnace (programat EP3010 Ivoclar Vivadent furnace), following predetermined parameters according to manufacturer of each material. IPS e.max and Amber groups were etched by hydrofluoric acid (9.5%) for 20 s while Tessera restorations were etched for 30 s, according to manufacturer instructions. The restorations were rinsed thoroughly with water for 20 s and dried with air stream. Silane coupling agent (Breeze, Pentron) was applied on the internal surface of overlays for 60s.A self-adhesive resin cement syringe (Breeze, Pentron, USA), Overlays were seated to the corresponding dies using finger pressure, followed by Light polymerization using LED light unit (LED-F, woodpecker, China) from a distance of 5 mm and allowed to self-cure for 6 min. After finishing and polishing samples, they were soaked in distilled water for 24 h before fracture testing.

### Fracture test

**Fig. 7:**
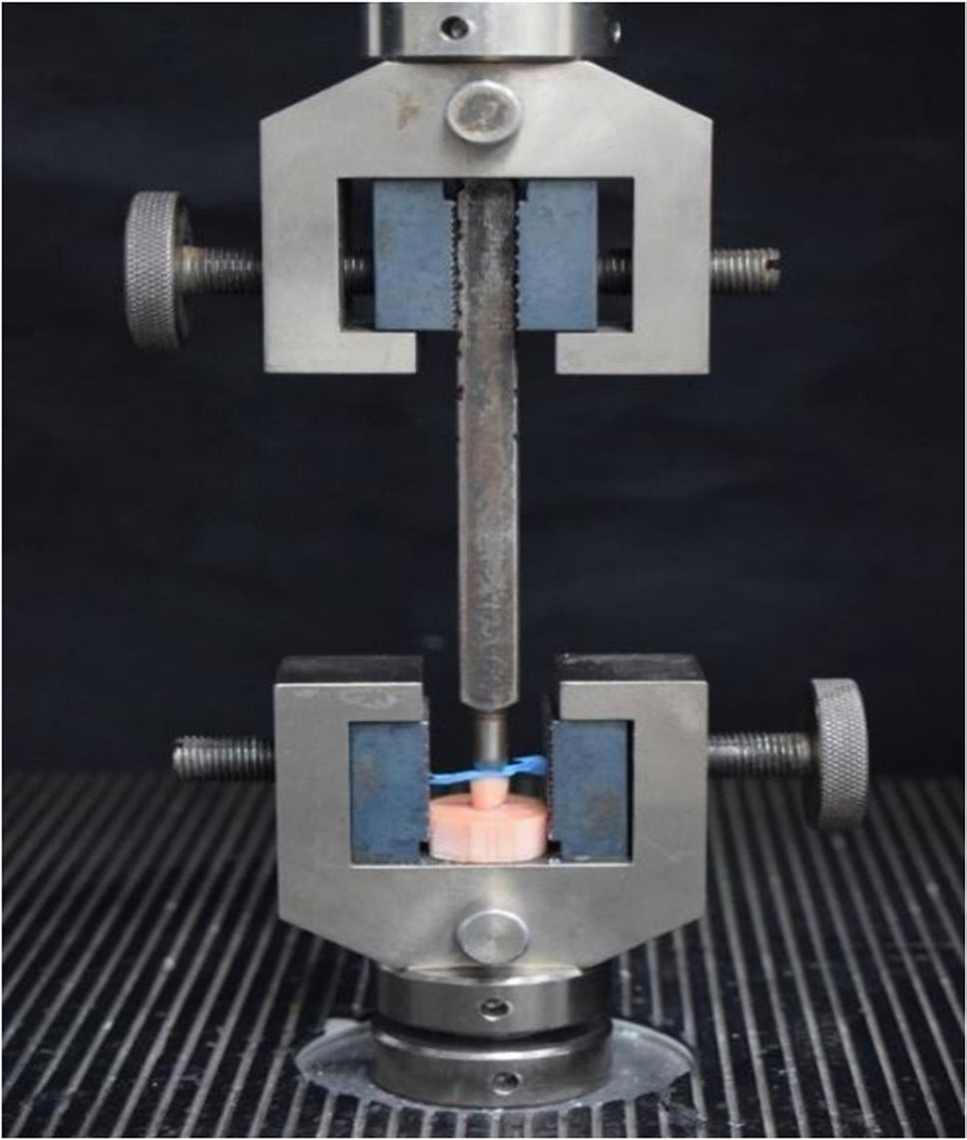
Testing fracture resistance.

All samples were individually mounted on a computer-controlled materials testing machine (Model 3345; Instron Industrial Products, Norwood, MA, USA) with a load cell of 5 kN and data were recorded using computer software (Instron^®^ Bluehill Lite Software). The compressive load was applied at the central fissure along long axis of the premolar using a 6-mm steel wedge at a 1 mm/min crosshead speed until fracture. To prevent localized stress concentration and achieve homogenous stress distribution. A 2-mm-thick urethane rubber sheet was inserted between the specimen and the metal wedge. The load at failure manifested by an audible crack and confirmed by a sharp drop at load-deflection curve recorded using computer software (Fig. 7).

### Scanning electron microscope observations

**Fig. 8:**
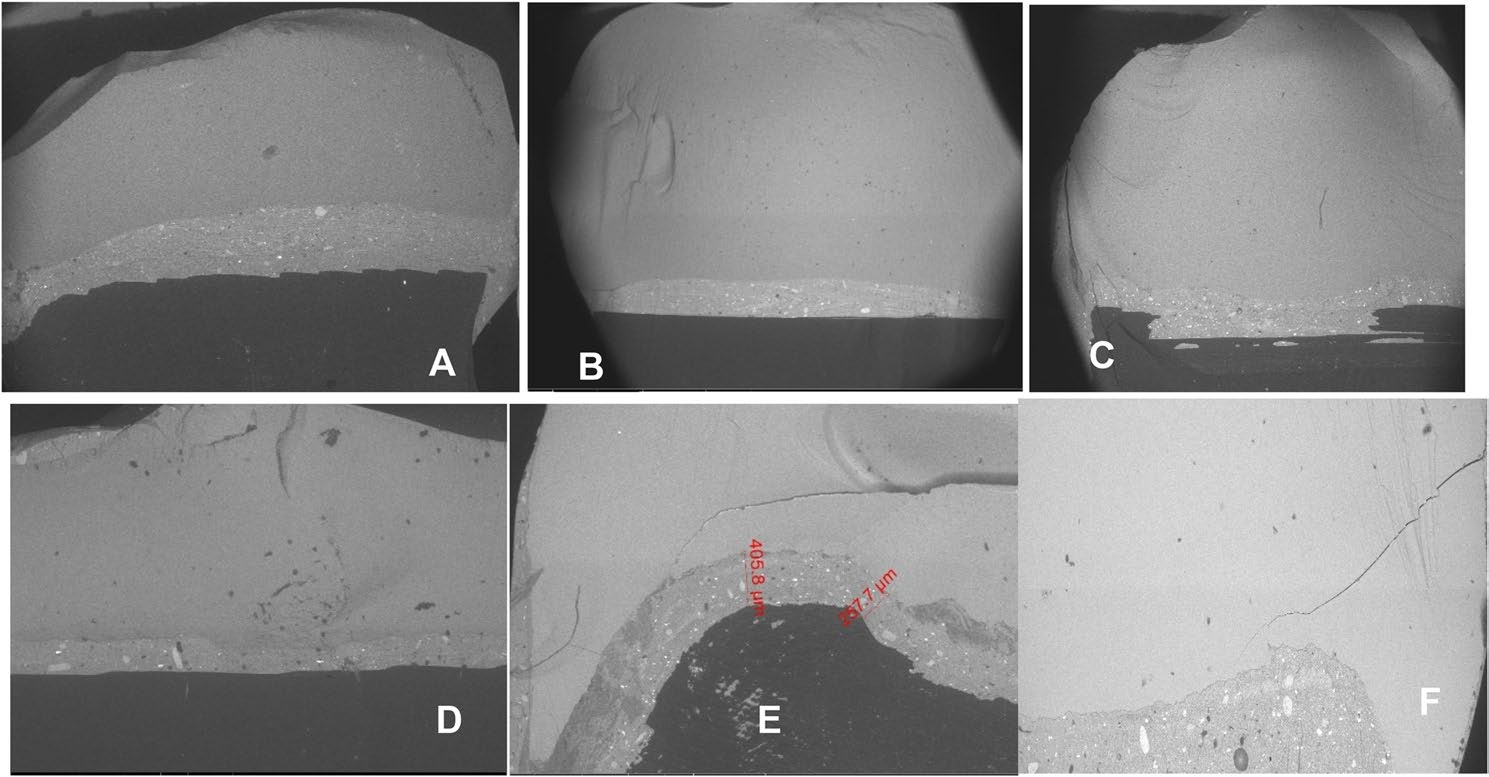
Representative SEM images of (AE-BE-AA-BA-AT-BT) alternative to (A-B-C-D-E-F) after fracture strength test

Field emission scanning electron microscopy (SEM; QUANTA, FEG 250, ELEC mi, Spain) of an accelerating voltage of 20 kV was used to visualize the cracked surface of restoration. The cracked surface of the restorations was examined in cross-section and analysed by a fractographic technique (Fig. 8).

### Failure mode and restorability index

After fracture resistance, samples were collected and inspected visually. Failure modes were identified and recorded based on tooth and/or and restoration damage using a modified restorability classification system [24, 25].

Grade I –Fracture of restoration only.

Grade II – Fracture of the crown of the tooth only.

Grade III – Fracture of restoration and the tooth crown.

Grade IV – Fracture of the root of the tooth.

Failure modes I–III are considered restorable, while mode IV is considered not restorable.

## Results

Design A showed statistically significantly higher fracture resistance values compared to Design B.

**Fig. 9:**
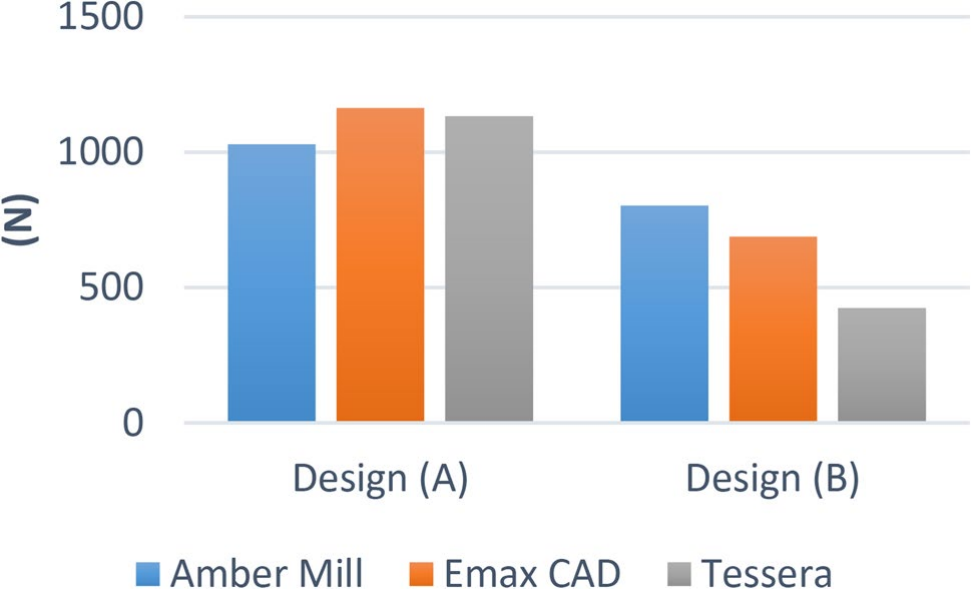
Bar chart showing average fracture resistance (N) for different materials within each design

**Fig. 10:**
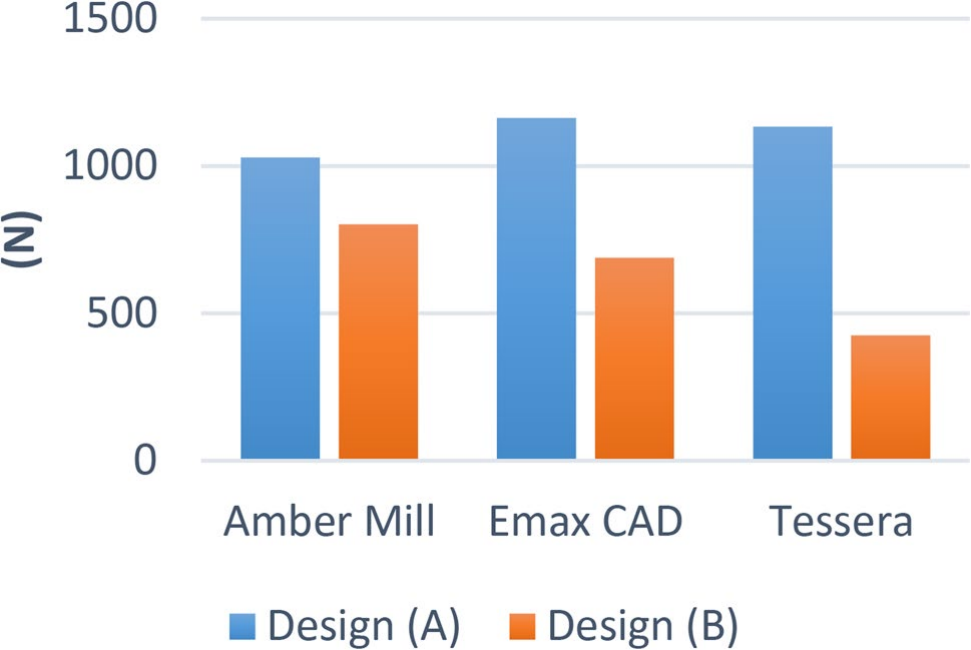
Bar chart showing average fracture resistance (N) for different designs within each material

There was no significant difference between the different groups (*p* = 0.363). Within design A, the highest value was found in IPS e.max CAD (1162.94 ± 243.08), followed by Tessera (1133.10 ± 150.63), while the lowest value was found at Amber Mill (1028.67 ± 240.22). While within design B, there was a significant difference between different groups (*p* < 0.001). The highest value was found in Amber Mill (802.26 ± 122.94), followed by IPS e-max CAD (688.16 ± 210.09), while the lowest value was found at Tessera (424.95 ± 38.85). Post hoc pairwise comparisons showed Tessera to have significantly lower value than other groups (*p* < 0.001) (Figs. 9 and 10).

### SEM observation results

Figure 8 illustrates SEM observations of the fractured surface of the restorations. Regardless of the type of restoration, many significant arrested lines were seen on the cracked surfaces at 20× magnification; however, CEREC Tessera designs had a far more complicated fracture pattern than the rest of overlay restorations. Wake hackles were more obvious at 100× magnification specially in IPS e-max CAD and Amber Mill restorations (whatever the design is), which are reliable markers of the direction of crack propagation. Yet, in CEREC Tessera crack lines were visible although the presence of hindering crystals for crack propagation.

### Failure mode and restorability analysis

BA showed grade III failure mode with the highest restorability possibility among other groups. BT showed the highest IV failure mode with zero restorability percentage (Table 1).

**Table 1:** Mode of fracture of experimental groups

Preparation design group	Mode of fracture				
	I	II	III	IV	Restorability%
AE	_	_	6	1	85.5%
BE	_	_	5	2	71.4%
AA	_	_	4	3	57.1%
BA	_	_	7	_	100%
AT	_	_	2	5	28.6%
BT	_	_	_	7	0%

## Discussion

The present comparative in vitro study aimed to determine whether the different preparation designs of overlay restoration using the newly introduced lithium disilicate ceramics (Amber Mill and Tessera) and the previously introduced lithium disilicate glass ceramic (IPS e-max CAD) affected their fracture resistance. The null hypothesis was rejected.

Lithium disilicate glass ceramic was used because of its adhesive properties [34] and its preservation of tooth structure [26]. Additionally, it can bear high occlusal stresses due to its slightly higher elastic modulus than enamel (95 GPa versus 84 GPa respectively) that builds up stresses leading to catastrophic breakdown. Cavity preparation exposes mostly dentin simulant that has a low modulus of elasticity, making it a reliable material for indirect restorations [27]. Besides, the superior aesthetic properties of lithium disilicate were considered [28].

Two preparation designs were selected in the present study; the first group (A design): 2 mm cavity depth was prepared in conjunction to 1.5 mm butt joint occlusal reduction. This design was chosen according to *Dhima et al.* [29] reporting that milled monolithic lithium disilicate crowns with 2.0- and 1.5-mm occlusal reduction require 17 and 15 times as many fatigue cycles to fail, respectively. There was a substantial difference between the thicknesses of 1.0 and 1.5 mm, which may be a crucial factor in the clinical performance of ceramic crowns. The second group (B design) includes 1.5 mm cavity depth with 2 mm butt joint occlusal reduction [1].

*Banks at al.* [30] suggested that the ideal occlusal box depth for inlay preparation should range from 1.5 mm at the fissure base to 2 mm at the cavity boundary. *Christian et al.* [31] reinforced these guidelines, reporting that fracture resistance values for Group A (natural tooth) and Group B (MOD with a 3 mm cavity depth) were relatively similar and significantly higher than those for Groups C and D (MOD with a 2 mm buccal or palatal reduction and a 1 mm cavity depth). The design of Group B was based on the findings of these two studies.

After preparing all samples, according to all previous studies, samples should be stored in distilled water for 24 h prior to test. Samples that tested in this manner, fractured in unusual pattern (cohesive fracture occurred in the die only) and at a lower value than expected. By contacting the manufacturer of resin material brand, they clarified that the resin is not suitable for storage in water after curing. So the rest of the samples were left at room temperature in dry conditions to lose all absorbed water, the data obtained from failed dies were rejected and new samples in return were prepared.

All specimens were subjected to occlusal loading using a metallic sphere to simulate natural force application on posterior teeth, enhancing the reliability of the results.

Regarding the results of our study, mean fracture load values for overlay designed premolar samples for A design were (1029 N, 1163 N, 1133 N) for AM, EX and CT respectively, whereas for B design were (802 N, 688 N, 425 N) for AM, EX and CT respectively. *Padma et al.* [32] Stated that the maximal biting force, employing an optical fiber-based bite force monitoring system, on the premolar is 464.5 N for men and 395.0 N for women. The highest bite force on the premolar was 422.9 N for men and 359.5 N for women, according to another related study [33].

Based on these results, overlay design B could be considered clinically acceptable in challenging cases. However, the use of CT as a restorative material with this cavity design is not recommended due to the lack of a safety margin between fracture resistance values and the maximum biting force according to the literature. On the other hand, cavity design A showed more favorable results and would be recommended as the standard preparation for all cases, especially in cases under higher occlusal loads. Comparing the fracture resistance values of the overlay samples for both designs: design A showed statistically significantly higher values than design B in the three materials. These findings are probably attributed to the more conservative design in means of 1.5 mm occlusal reduction with a 2 mm intra-coronal extension, further enhancing the fracture resistance arm, without compromising the dentin bridge protecting the pulp.

Regarding the effect of LDC materials on the fracture resistance, In design A: there was no significant difference in fracture resistance using different LDC materials and all of EX, AM, CT were within acceptable clinical range. However, in design B: AM showed significantly high fracture resistance followed by EX then CT.

This could be explained by the fact that AM is a nanocrystalline LDC material which undergoes heat treatment leading to increasing crystal size and density, strengthening it’s mechanical qualities. In addition to its highly stable edges with low chipping occurrence during milling proving that Amber Mill is one of the best machinable lithium disilicate blocks for CAD/CAM system. These assumptions were supported by *Jurado et al.* [34].

Meanwhile CT showed the lowest fracture resistance probably resulting from the uneven distribution of virgilite and lithium disilicate crystals throughout the glassy matrix. Another possibility is that the crystal formation was hindered by the low firing temperature of (760 °C) in comparison to (840 °C) for IPS e.max CAD leading to loose distribution of Lithium Disilicate and Virgilite crystals in the matrix. This has been observed as a cohesive failure within the matrix, contributing to the low strength properties even after the addition of Virgilite crystals. All these factors ultimately explain CT’s inability to function cohesively [35].

Typodont teeth were used in this study to perform a simulation of the clinical preparation for overlays, natural teeth present great variation considering the age, individual structures, and time of storage, making the standardization of the tests very challenging [36]. A CNC machine was used to prepare teeth in uniform, non-human error and precise dimensions following accurate design. Resin dies were selected as a replacement for dentin since the material has a tensile strength of 61 MPa, which is within the range of dentin tensile strength (44.4 to 97.8 MPa) [37, 38].

Fracture resistance and flexural strength are commonly used to describe material strength and predict clinical success or failure. However, the strength of ceramic is a complex parameter that cannot be fully described by a single value. The maximum flexural strength varies greatly from sample to another according to distribution of flaws present in the samples. When flaws are consistent and evenly distributed, the flexural strength of a sample will behave more consistently than when flaws are clustered inconsistently. Fracture resistance might not correspond to respective uniaxial flexural strength of the material because mechanical behavior of the complexity of restored tooth, adhesive system and restoration can’t be predicted. Thus, relying on fracture resistance as in in-vitro testing to the mechanical properties is more clinically relevant knowing that samples used are anatomical restorations rather than plain discs [39].

Factors associated with clinical success of ceramic restorations appear to be not only related to cavity preparation designs, but also to the choice of ceramic material. Ceramic materials with more favorable mechanical properties have the potential to function more effectively in clinical situations [40].

While the findings of this study provide valuable insights, certain methodological considerations should be noted. The use of a non–water-resistant die may not fully mirror the intraoral environment, where moisture exposure is inevitable; employing a water-resistant die in future research could enhance clinical relevance. In addition, the experimental design did not include a periodontal ligament (PDL) analogue, which in vivo serves a shock-absorbing role and may influence the strength and fracture pattern of teeth during mastication [41]. Future studies incorporating a PDL simulation may therefore provide a more comprehensive representation of clinical conditions.

### Recommendations

- More in vitro and in vivo studies should be conducted to validate the reliability of our results and ensure the long-term clinical serviceability of overlay restorations.
- 3D printing and CAD technology need to be further evaluated for measurement accuracies.
- 1.5 mm and 2 mm occlusal reduction doesn’t affect fracture resistance, it’s more important to have cavity depth not less than 2 mm.

## Conclusion

1. The design of the preparation significantly influences the fracture resistance of overlay restorations, while the type of material has a minor impact.
2. Design A with a deeper cavity (2 mm) in relation to occlusal reduction (1.5 mm) has higher fracture resistance values than design B using lithium disilicate overlay preparation design.
3. Despite the inclusion of additional virgilite crystals in CT, they had no significant effect on fracture resistance.

## Data Availability

The datasets used and/or analyzed during the current study available from corresponding author on reasonable request.

## Abbreviations

CAD/CAM: Computer aided design/ computer aided machining
CNC: Computerized numerical control
CT: Cerec Tessera
AE: Design (A) E.MAX
BE: Design (B) E.MAX
AA: Design (A) Amber Mill
BA: Design (B) Amber Mill
AT: Design (A) Tessera
BT: Design (B)Tessera
LDC: Lithium disilicate ceramics
